# A CD30-positive variant of intravascular large B-cell lymphoma presenting as diffuse interstitial lung disease and generalized lymphadenopathy: a case report

**DOI:** 10.3389/fmed.2026.1790176

**Published:** 2026-02-27

**Authors:** Lei Wang, Yongdeng Cai, Mengyao Wang, Cui Gao, Jianni Zhu, Guangwei Xue, Zheng Dong, Changsheng Ge, Wei Zhang, Zongfang Li, Zongtao Liu, Yunqing Chen, Cailing Du, Changjiang Li, Yajing Liang, Lu Wang, Baomei Mi

**Affiliations:** 1 Department of Respiratory and Critical Care Medicine, Rizhao Hospital of Traditional Chinese Medicine, Rizhao, China; 2 Department of Respiratory, Linyi Central Hospital, Linyi, China; 3 Department of Pathology, The Affiliated Hospital of Qingdao University, Qingdao, China; 4 Department of Radiology, Rizhao Hospital of Traditional Chinese Medicine, Rizhao, China; 5 Department of Pathology, Rizhao Hospital of Traditional Chinese Medicine, Rizhao, China

**Keywords:** CD30, hypersensitivity pneumonitis, interstitial pneumonia, intravascular large B-cell lymphoma, lymphoproliferative disorder

## Abstract

**Introduction:**

Intravascular large B-cell lymphoma (IVLBCL) is a rare and aggressive malignancy defined by the proliferation of neoplastic B-cells within the vascular lumen. While the disease can affect multiple organs, commonly manifesting as skin lesions, neurological deficits, or hepatosplenomegaly, it usually spares the lymph nodes and rarely presents as diffuse interstitial lung disease (DILD). We report a 62-year-old man admitted with recurrent fever and progressive dyspnea. Chest computed tomography revealed extensive bilateral interstitial lung disease, with ultrasonography showing generalized lymphadenopathy with preserved architecture. Although initial therapy led to a favorable response, the patient subsequently relapsed. Lymph node biopsy confirmed IVLBCL, characterized by intravascular and intrasinusoidal lymphoma cell infiltration with well-preserved nodal architecture. Notably, the tumor cells showed diffuse strong CD30 expression (~70%), a rare finding potentially linked to the unusual clinical presentation.

**Conclusion:**

This case demonstrates that IVLBCL can present with diffuse interstitial lung disease and generalized lymphadenopathy, expanding its recognized phenotypic spectrum. It may represent a variant with CD30-mediated nodal homing and immune activation.

## 1 Introduction

Intravascular large B-cell lymphoma (IVLBCL) is a rare disease characterized by the accumulation of a large number of neoplastic B cells in the vascular lumen (1). The disease can affect any organ, and clinical manifestations include constitutional symptoms, skin lesions, stroke, focal neurological dysfunction, dyspnea, hepatosplenomegaly, and splenic infarction (2). However, the lymph nodes are usually spared (3). Cases first manifesting in the lungs are rare and difficult to diagnose (4), and the overall prognosis remains poor (5).

### 1.1 Case information

**Figure 1:**
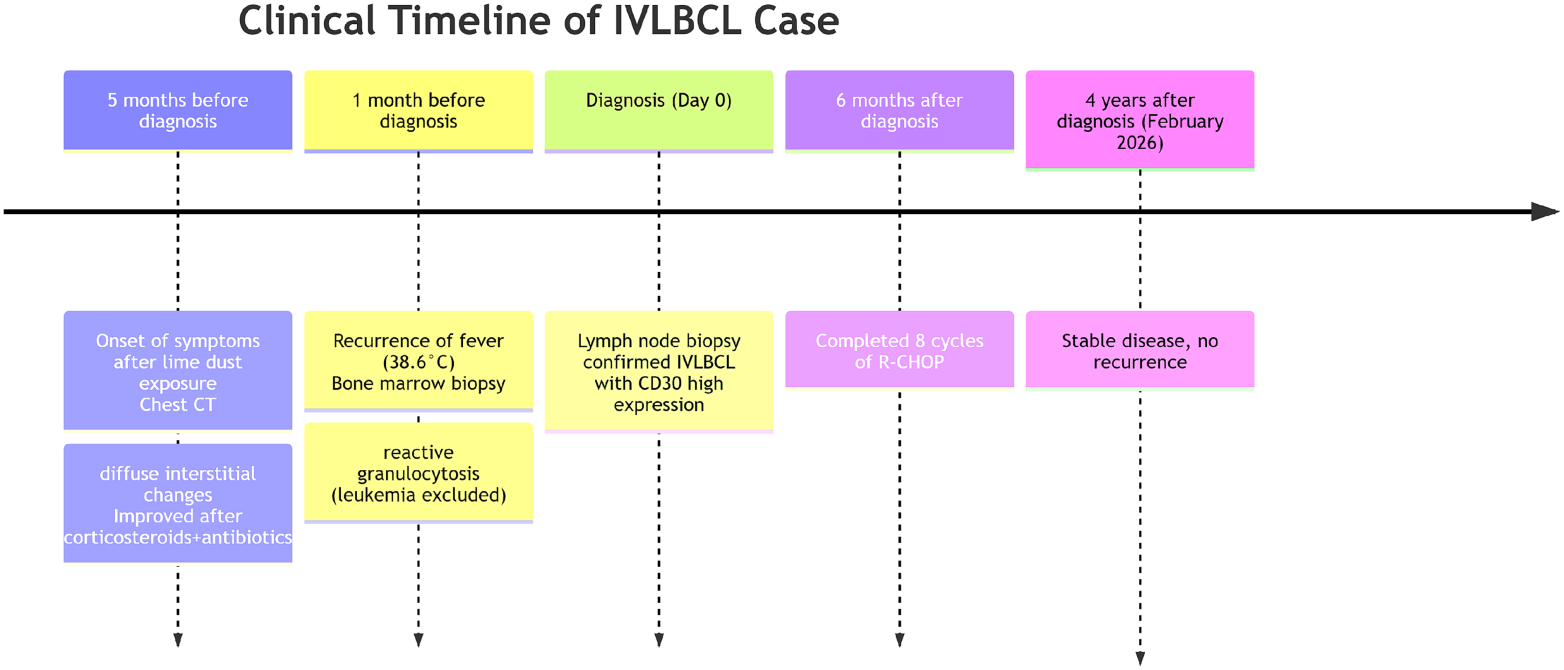
Timeline of clinical course. The patient initially presented 5 months before diagnosis with respiratory symptoms following occupational lime powder exposure. After transient improvement with empirical therapy, he relapsed 1 month prior to admission. Diagnosis of IVLBCL was established by lymph node biopsy at admission. The patient completed 8 cycles of R-CHOP chemotherapy over the subsequent 6 months and remained disease-free at the last follow-up in February 2026.

The clinical timeline is summarized in Figure 1, illustrating the relapsing–remitting course. The detailed clinical course is as follows.

**Figure 2:**
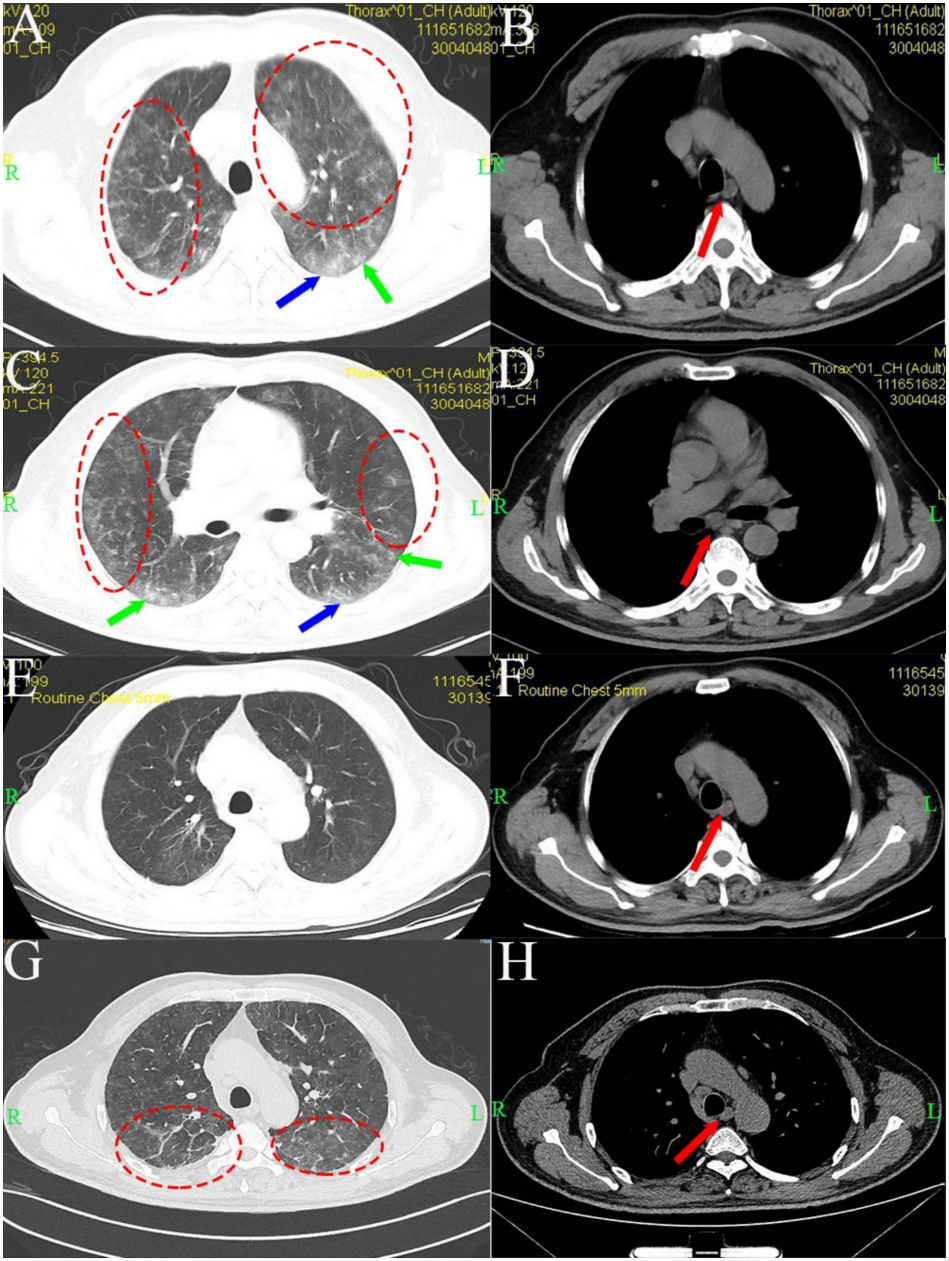
Chest CT imaging showing treatment response and subsequent recurrence. Initial CT **(A–D)** demonstrates diffuse bilateral ground-glass opacities, nodules, and peripheral reticulations (most prominent in the lower lungs), accompanied by mediastinal lymphadenopathy. Follow-up CT after a 10-day course of methylprednisolone and antimicrobial therapy shows marked resolution of the pulmonary lesions **(E,F)**. A subsequent HRCT confirms recurrence, with extensive ground-glass/reticular opacities and persistent mediastinal lymphadenopathy **(G,H)**. Red dashed circles indicate ground-glass opacities; green arrows indicate nodular opacities; blue arrows indicate reticular opacities; red arrows indicate lymphadenopathy.

A 62-year-old man with no history of smoking, alcohol use, or psychiatric disorders, and no relevant family history, presented with 5 months of cough and chest tightness that began after occupational exposure to lime powder. At initial presentation 5 months prior to admission, chest computed tomography (CT) with a slice thickness of 5 mm from a local hospital revealed diffuse bilateral ground-glass opacities and nodules, as well as peripheral reticulations most prominent in the bilateral lower lungs, in addition to mediastinal lymphadenopathy (Figures 2A–D). Laboratory tests at that time showed elevated C-reactive protein (88.2 mg/L) and erythrocyte sedimentation rate (68 mm/h), hypoxemia (PaO₂ 58 mmHg; SpO₂ 91% on room air), and lymphopenia (14.9%). Complete blood count, including hemoglobin and platelets, was normal, as were serum immunoglobulin levels (IgG 10.40 g/L, IgA 1.97 g/L, IgM 0.99 g/L). Serologies for mycoplasma, fungal antigens (G/GM tests), and rheumatic disease panels were negative. Given the exposure history and imaging findings, hypersensitivity pneumonitis with possible bacterial co-infection was initially suspected. After a 10-day course of methylprednisolone and antimicrobial therapy with intravenous mezlocillin and levofloxacin, his symptoms improved significantly, and a follow-up CT with the same slice thickness showed marked resolution of the pulmonary lesions (Figures 2E,F). One month before admission to our hospital, the patient developed a fever of 38.6 °C. A bone marrow biopsy was performed to rule out leukemia, which revealed reactive granulocytosis.

**Figure 3:**
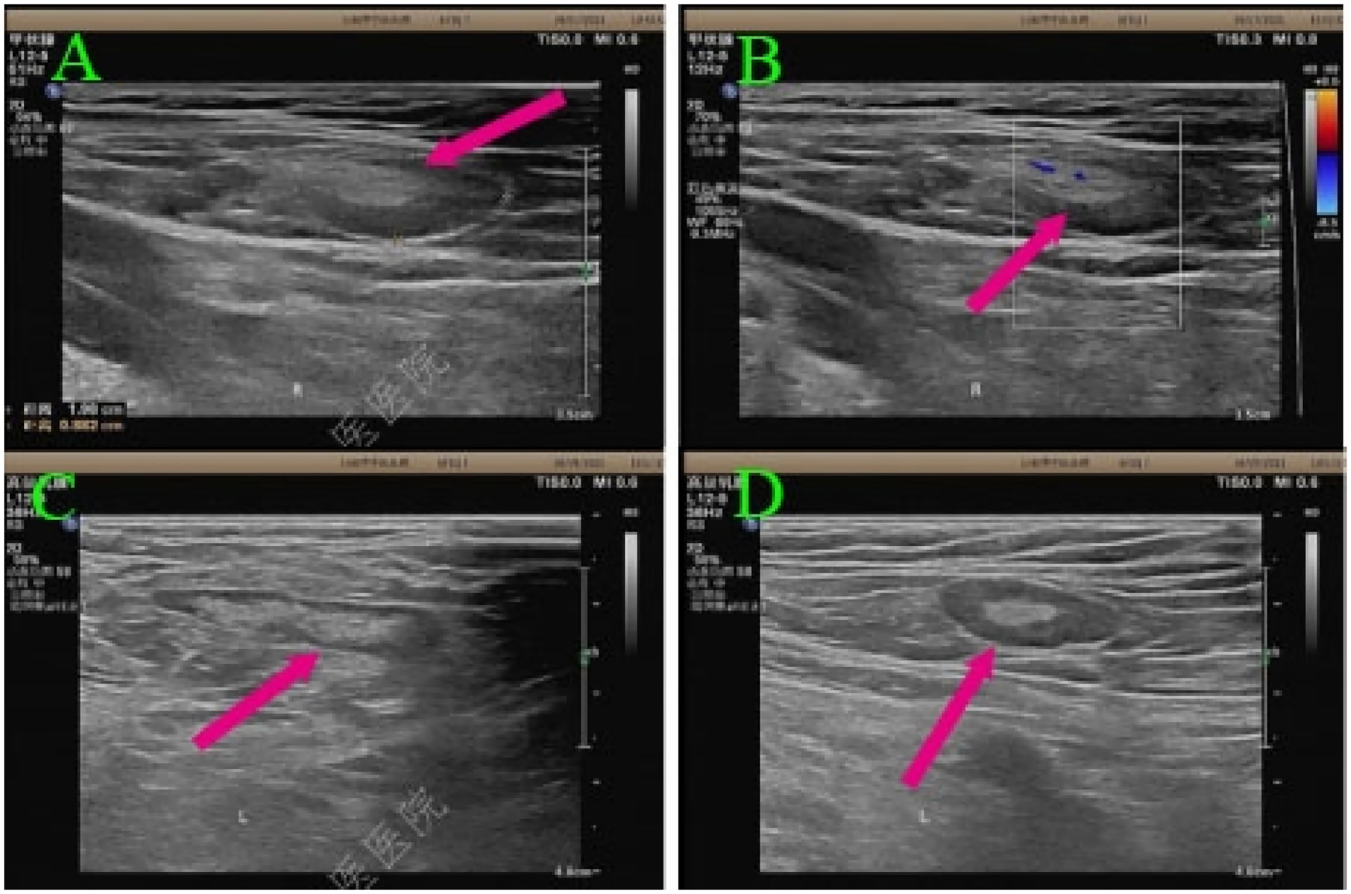
Ultrasonography reveals enlarged lymph nodes in the cervical **(A)**, axillary **(B)**, and inguinal **(C,D)** regions, with preserved nodal architecture, characterized by regular morphology and visible echogenic hilum, with spot-like and linear blood flow signals within the lymph nodes.

**Figure 4:**
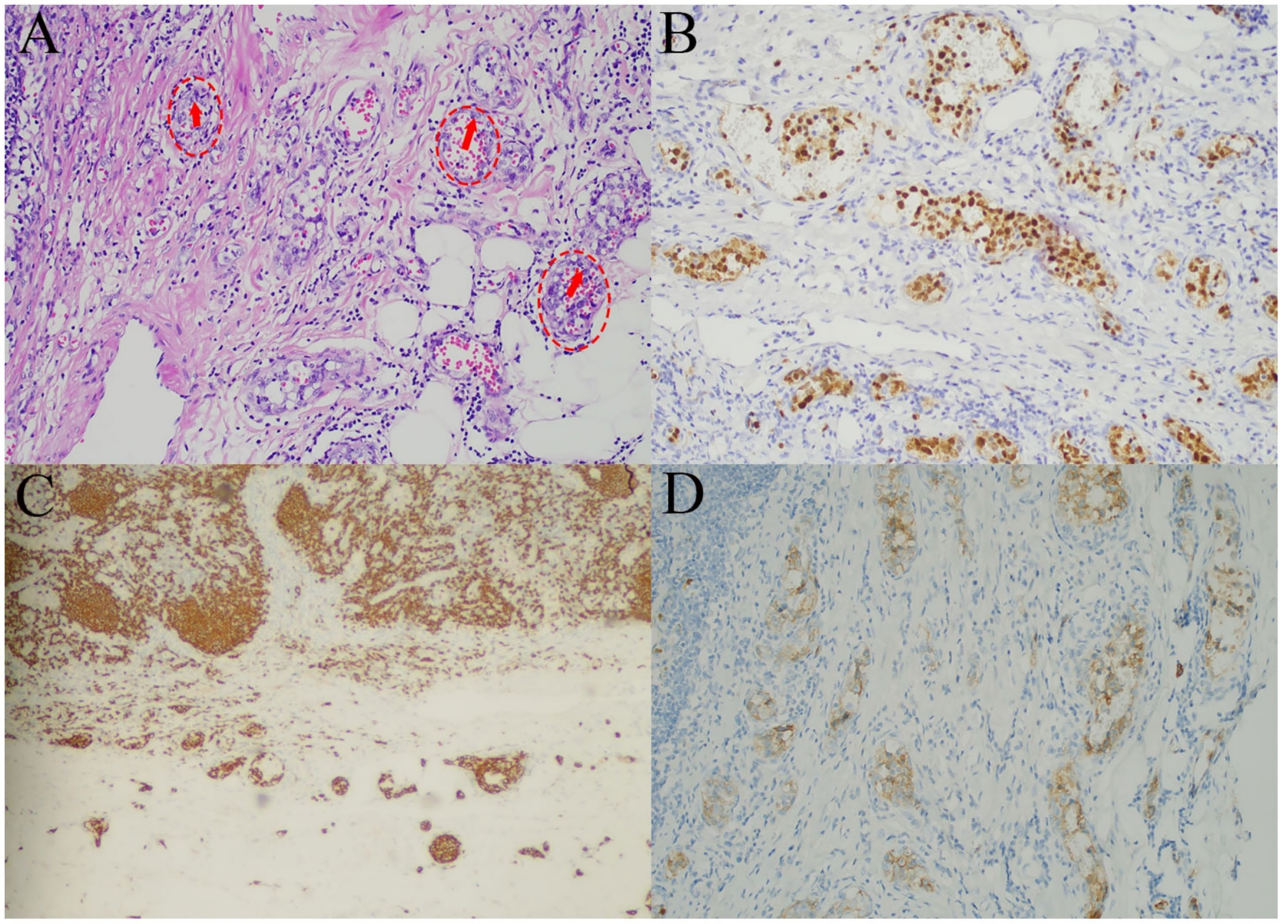
Histopathological and immunohistochemical features of the lymph node biopsy. H & E section of lymph node reveals the large B-cell lymphoma cells within vascular lumina of the lymph node parenchyma and the surrounding adipose tissue **(A)** (100x magnification). Immunohistochemistry shows the tumor cells are positive for MUM1 **(B)**, CD20 **(C)**, and CD30 **(D)** (100x magnification). Red arrows indicate lymphoma cells; red dashed circles outline vascular walls.

The patient was admitted to our hospital for recurrent fever. On admission, vital signs were stable except for a fever of 39.4 °C. Arterial blood gas on room air revealed hypoxemia and respiratory alkalosis. Chest auscultation revealed slightly coarse breath sounds without rales. Serum ferritin was elevated (697.70 ng/mL), while all other tumor markers (AFP, CEA, CA19-9, CA125, NSE) and autoimmune antibodies were within normal limits. Screening for hepatitis B, hepatitis C, syphilis, and HIV was negative, as were tests for SARS-CoV-2, cytomegalovirus, respiratory syncytial virus, and other viral pathogens. Chest HRCT showed extensive ground-glass and reticular opacities and mediastinal lymphadenopathy (Figures 2G,H), with a subsequent contrast-enhanced CT revealing no definite mass. Bronchoalveolar lavage (BAL) analysis revealed a neutrophilic-predominant profile with profound lymphocytopenia (2%), which argued against hypersensitivity pneumonitis. Microbiological culture of the BAL fluid grew *Klebsiella pneumoniae*. The chronic radiographic pattern of reticulation argued against acute viral pneumonia, and negative serologies ruled out connective tissue disease-associated ILD. Transbronchial lung biopsy showed alveolar septal infiltration by atypical lymphoid cells, some intravascular. Immunohistochemical staining showed that the tumor cells were positive for CD20, CD68, LCA, and CD34(vascular+), while negative for PAS and EMA. This immunophenotype confirmed a B-cell lineage lymphoma. However, due to the limited tissue obtained, a definitive subclassification could not be made. Therefore, additional evidence was sought. Ultrasonography reveals enlarged lymph nodes in the cervical, axillary, and inguinal regions, with preserved nodal architecture, characterized by regular morphology and visible echogenic hilum, with spot-like and linear blood flow signals within the lymph nodes (Figures 3A–D). Histopathological examination of the lymph node biopsy revealed reactive lymphoid hyperplasia with preserved nodal architecture, along with accumulation of lymphoma cells within the sinuses, blood vessels, and surrounding adipose tissue vessels (Figure 4A), confirming the diagnosis of IVLBCL. Immunohistochemical staining showed the tumor cells to be positive for MUM1 (Figure 4B), CD20 (Figure 4C), CD30 (~70%) (Figure 4D), CD34(vascular+), CD38 (scattered+) and weakly for Bcl-6, with a high Ki-67 index (~90%), but negative for CD10, Bcl-2, ALK-D5F3, and Epstein–Barr encoded RNA (EBER). Notably, CD3 and CD5 highlighted background T-cells, c-myc showed low positivity (~5%), P53 showed individual positivity, and CD21 outlined the follicular dendritic cell network. These histopathological features established the definitive diagnosis of intravascular large B-cell lymphoma.

### 1.2 Outcome

The patient received antibiotics, corticosteroids, and supportive care during his initial hospitalization. After discharge, he completed eight cycles of the rituximab, cyclophosphamide, pirarubicin, vincristine and prednisone (R-CHOP) chemotherapy and remained stable through February 2026.

## 2 Discussion

### 2.1 Clinical and radiological features of IVLBCL

IVLBCL is a rare extranodal large B-cell lymphoma defined by selective intravascular tumor growth (6). Although its pathogenesis is not fully understood, it may be linked to adhesion molecule deficiencies (e.g., CD18, CD29, CD54) (7, 8) and chromosomal abnormalities. IVLBCL is a multisystem disorder that typically presents with fever, skin lesions, neurological signs, hepatosplenomegaly, and pancytopenia (3, 9), while characteristically sparing peripheral lymph nodes (3, 10) and rarely presenting with pulmonary symptoms (11). Laboratory studies frequently show elevated lactate dehydrogenase (LDH) and erythrocyte sedimentation rate (ESR) (12). Radiologically, pulmonary involvement most characteristically presents as interlobular septal thickening, centrilobular nodules, and ground-glass opacities (GGO) (13, 14). These lesions typically exhibit intense fluorodeoxyglucose avidity on positron emission tomography, which can be evident even in the absence of correlative CT abnormalities (15). In the present case, the early chest CT demonstrated diffuse interstitial pneumonia, including interlobular septal thickening and both nodular and patchy ground-glass opacities, consistent with the described spectrum.

### 2.2 Diagnosis of IVLBCL

The diagnosis of IVLBCL requires biopsy demonstrating proliferating lymphoma cells within vascular lumina (15), coupled with immunohistochemical analysis revealing positive staining for one or more pan-B-cell markers (16). Notably, sinusoidal growth is a well-known feature of IVLBCL (17). In the present case, histopathological examination of the lymph node biopsy showed accumulation of lymphoma cells within sinuses, blood vessels, and perivascular adipose tissue, alongside reactive lymphoid hyperplasia with preserved nodal architecture. Given that IVLBCL is defined by the exclusive confinement of tumor cells to vascular and sinusoidal spaces without a solid component, these findings confirmed the diagnosis.

### 2.3 Interstitial lung disease mimics

Our patient presented with fever and dyspnea after lime dust exposure, a highly misleading picture that initially suggested hypersensitivity pneumonitis. This diagnosis was, however, excluded by the finding of profound lymphocytopenia (2%) on bronchoalveolar lavage (BAL) analysis. The clinical, imaging, and laboratory findings in this case—including respiratory symptoms, ground-glass opacities, nodules, reticulation, and systemic lymphadenopathy—overlap with those of granulomatous-lymphocytic interstitial lung disease (GLILD), a multisystem disorder (18). Crucially, however, the patient’s serum immunoglobulin levels at the time of onset, failing to meet the essential diagnostic criteria for common variable immunodeficiency (CVID) (19). Moreover, pulmonary histopathology revealed markedly atypical lymphoid cells with predominantly intravascular growth and sinusoidal infiltration. These findings, together with the nodal immunophenotype (CD20+, MUM1+) and a high proliferative index (Ki-67 ~ 90%), unequivocally indicate a neoplastic process that is incompatible with the pathological features of GLILD. The lack of diffuse alveolar damage and non-necrotizing granulomas on biopsy argued against acute interstitial pneumonia and sarcoidosis, respectively. Viral infections were ruled out based on the early reticular pattern on imaging, a neutrophilic-predominant BAL profile, and a chronic clinical course. Autoimmune serologies were also negative, effectively ruling out connective tissue disease-associated interstitial lung disease (ILD), and contrast-enhanced chest CT showed no mass suggestive of carcinoma.

### 2.4 Intravascular lymphoma mimics

An increasing number of reports of other intravascular/intralymphatic lymphomas, such as Epstein–Barr virus (EBV)-positive NK/T-cell lymphoma or ALK-negative anaplastic large cell lymphoma (ALCL) (3), necessitate careful pathologic distinction. EBV-positive NK/T-cell lymphoma was ruled out by negative EBER *in situ* hybridization. ALK-negative intralymphatic ALCL was excluded due to a lack of T-cell markers, with CD3 and CD5 immunostaining selectively highlighting background T-cells.

### 2.5 Differential diagnosis of sinusoidal infiltration

Another notable feature of this case is the presence of sinusoidal infiltration in addition to the characteristic peripheral nodal intravascular growth—a pattern well-recognized in inborn errors of immunity (IEI) [e.g., autoimmune lymphoproliferative syndrome (ALPS) and immunodeficiency-associated lymphoid proliferations (LPD)], Castleman disease, and various lymphomas (e.g., ALCL, EBV + T/NK-cell lymphomas, anaplastic DLBCL, and IVLBCL) (20), with rare reports also in Hodgkin lymphoma (21). The absence of clinical or laboratory evidence of immunodeficiency, lack of polymorphic histology, negative EBV, and predominantly exclusive intravascular growth argue against the diagnosis of polymorphic immunodeficiency-associated LPD. Castleman disease was excluded by the absence of hyaline vascular follicles or interfollicular sheet-like plasma cell proliferation on biopsy, along with normal hemoglobin, platelet count, and immunoglobulin levels—all inconsistent with this entity. Furthermore, although the anaplastic variant of DLBCL, not otherwise specified (DLBCL-NOS), can share a sinusoidal growth pattern, its diagnosis requires a solid tumor mass or diffuse infiltration, whereas IVLBCL is defined by the exclusive confinement of tumor cells to vascular and sinusoidal lumina without a solid component. The purely intravascular/sinusoidal growth observed in this case is the defining hallmark of IVLBCL and definitively excludes anaplastic DLBCL-NOS.

### 2.6 Differential diagnosis of lymphadenopathy

This patient presented with systemic lymphadenopathy, possibly related to viral infections or immune deficiency and dysregulation-associated lymphoproliferative disorders and lymphomas (IDD-LPDs). Indeed, distinguishing reactive from neoplastic proliferations in IEI is a notoriously challenging diagnostic gray zone (22)—31% of cases initially diagnosed as lymphoma were reclassified as reactive upon expert central review (23). We acknowledge that the absence of germline sequencing cannot absolutely exclude an underlying IEI; nevertheless, using available clinical, laboratory, and pathological parameters, we have systematically excluded all major IEI-LPD entities. ALPS typically presents at age 5–44 years with intact nodal architecture, paracortical hyperplasia, and sinus histiocytosis (22); this entity is excluded in our patient based on elderly age, lack of paracortical hyperplasia, exclusive intravascular lymphoma growth, normal immunoglobulins, normal platelets, and negative family history. Normal serum IgG (10.40 g/L) excludes CVID; negative EBV *in situ* hybridization rules out EBV-driven IEI; normal platelet count alone excludes Wiskott-Aldrich syndrome; adult onset, absence of syndromic features, and immunocompetent status exclude syndromic combined immunodeficiencies; and DNA repair disorders—defined by childhood-onset neurologic/syndromic features, recurrent infections, hypogammaglobulinemia, and elevated alpha-fetoprotein (AFP)—are clinically excluded by our patient’s adult onset, absence of such features, normal immunoglobulins, and negative EBV status. Bone marrow and peripheral blood were unremarkable, excluding primary hematologic malignancies with secondary nodal involvement. Furthermore, negative serologies for human immunodeficiency virus (HIV), severe acute respiratory syndrome coronavirus 2 (SARS-CoV-2), and cytomegalovirus (CMV) further exclude viral infection-associated reactive lymphadenopathy. Integrating all clinical, laboratory, and pathological findings—particularly the hallmark exclusive and dominant intravascular growth within nodal peripheral vessels—this case is best classified as primary IVLBCL in an immunocompetent host.

### 2.7 Differential diagnosis of CD30-positive diseases

A distinctive feature of this case is the diffuse and strong CD30 expression (~70%) by the neoplastic cells. CD30, a member of the tumor necrosis factor receptor superfamily (TNFRSF8), is an activation marker of B and T cells with pleiotropic, cell type-specific effects on proliferation and apoptosis. Its expression has been documented across a broad spectrum of conditions—including viral infections, immune dysregulation, and various B- and T-cell lymphomas [e.g., ALCL, peripheral T-cell lymphoma (PTCL) subtypes, NK-cell neoplasms (24), classic Hodgkin lymphoma, primary mediastinal large B-cell lymphoma (PMBL), the anaplastic variant of DLBCL-NOS, and mycosis fungoides (3, 25)], as well as non-hematologic neoplasms (e.g., aggressive systemic mastocytosis, embryonal carcinoma). Thus, CD30 positivity alone does not define lineage or disease entity. The diagnosis of PMBL is not favored clinically due to the patient’s older age, absence of a dominant anterior mediastinal mass, and the presence of intact nodal architecture. Histopathologically, CD3 and CD5 immunostaining highlighted only background small T-lymphocytes, with no expression on neoplastic cells; together with the cytomorphology of the tumor cells, this finding excludes mycosis fungoides, specific subtypes of PTCL, NK-cell neoplasms, and other T-cell lymphomas. The cytomorphology also does not support classic Hodgkin lymphoma. Aggressive systemic mastocytosis was excluded by the absence of mast cell morphology and unremarkable bone marrow examination, while embryonal carcinoma was ruled out by normal serum AFP levels and the absence of testicular enlargement or a primary gonadal/extragonadal mass.

### 2.8 Potential mechanisms of lymphadenopathy and pulmonary involvement

Generalized lymphadenopathy in IVLBCL is unusual. The preserved nodal architecture observed on both ultrasonography and histopathology, along with corresponding intrasinusoidal lymphoma cell accumulation, suggests that obstructive sinus dilatation mediates lymph node enlargement. This unusual nodal tropism may be linked to the tumor’s distinct immunophenotype. CD30 is frequently highly expressed in ALCL, classic Hodgkin lymphoma, and anaplastic DLBCL-NOS, all of which commonly present with systemic lymphadenopathy (3). Given that Hodgkin lymphoma and IVLBCL utilizes CD30 to activate nuclear factor kappa-B (NF-κB) (25, 26), and that physiological CD30^+^ B cells localize to germinal centers (27) with homing receptor-associated activation profiles (28), we hypothesize that high CD30 expression in this case may drive lymphadenopathy by co-opting normal CD30^+^ B-cell homing programs via NF-κB. Similarly, murine studies show sustained B cell-intrinsic CD30 signaling induces B-cell expansion and lymphomagenesis in immunocompetent contexts, accelerated by C-X-C chemokine receptor type 4 (CXCR4) upregulation (29, 30). Thus, immune activation pathways may also contribute. Furthermore, CD30–Janus kinase-signal transducer and activator of transcription 6 (JAK-STAT6) and CD30–integrin axes in the pulmonary microenvironment may link to concurrent interstitial lung disease (31). Therefore, this case may represent an IVLBCL variant with CD30 positivity, nodal homing propensity, and/or potential immune activation, expanding the clinicopathological spectrum of IVLBCL.

### 2.9 Treatment and prognosis

Approximately 80% of IVLBCL patients are treated with systemic corticosteroid-containing chemotherapy, such as cyclophosphamide, doxorubicin, vincristine, and prednisone (CHOP) or rituximab plus CHOP (R-CHOP) (32). Early anthracycline-based therapy or rituximab inclusion improves efficacy and prognosis (12), with 3-year overall survival reaching 60–81% (3). Outcomes, however, vary significantly based on cell-of-origin subtype and involved organs.

### 2.10 Study limitations

This first reported case of CD30 + IVLBCL mimicking occupational lung disease, GLILD, and other lymphoproliferative disorders expands its clinicopathological spectrum. However, the absence of PET-CT precludes definitive primary site identification, and the lack of germline sequencing precludes definitive exclusion of an underlying IEI with incomplete penetrance.

## 3 Conclusion

CD30-positive variant of IVLBCL may present with DILD and lymphadenopathy, expanding its recognized phenotypic spectrum. In evaluating atypical interstitial lung disease, clinicians should consider intravascular large B-cell lymphoma—even with exposure history or uncommon systemic lymphadenopathy—prioritize definitive biopsy, and employ multidisciplinary assessment to distinguish this rare malignancy.

## 4 Patient perspective

I was diagnosed with a very rare vascular lymphoma after a long diagnostic journey. While the process was complex, I feel fortunate to have reached a definitive diagnosis and received effective treatment. My condition is now stable, thanks to the medical team’s persistent care.

## Data Availability

The raw data supporting the conclusions of this article will be made available by the authors, without undue reservation.
