# Supplementary figures and images for: A CD30-positive variant of intravascular large B-cell lymphoma presenting as diffuse interstitial lung disease and generalized lymphadenopathy: a case report
Source: Front Med (Lausanne). 2026 Feb 27;13:1790176. doi: 10.3389/fmed.2026.1790176 (PMC12982330; doi:10.3389/fmed.2026.1790176)

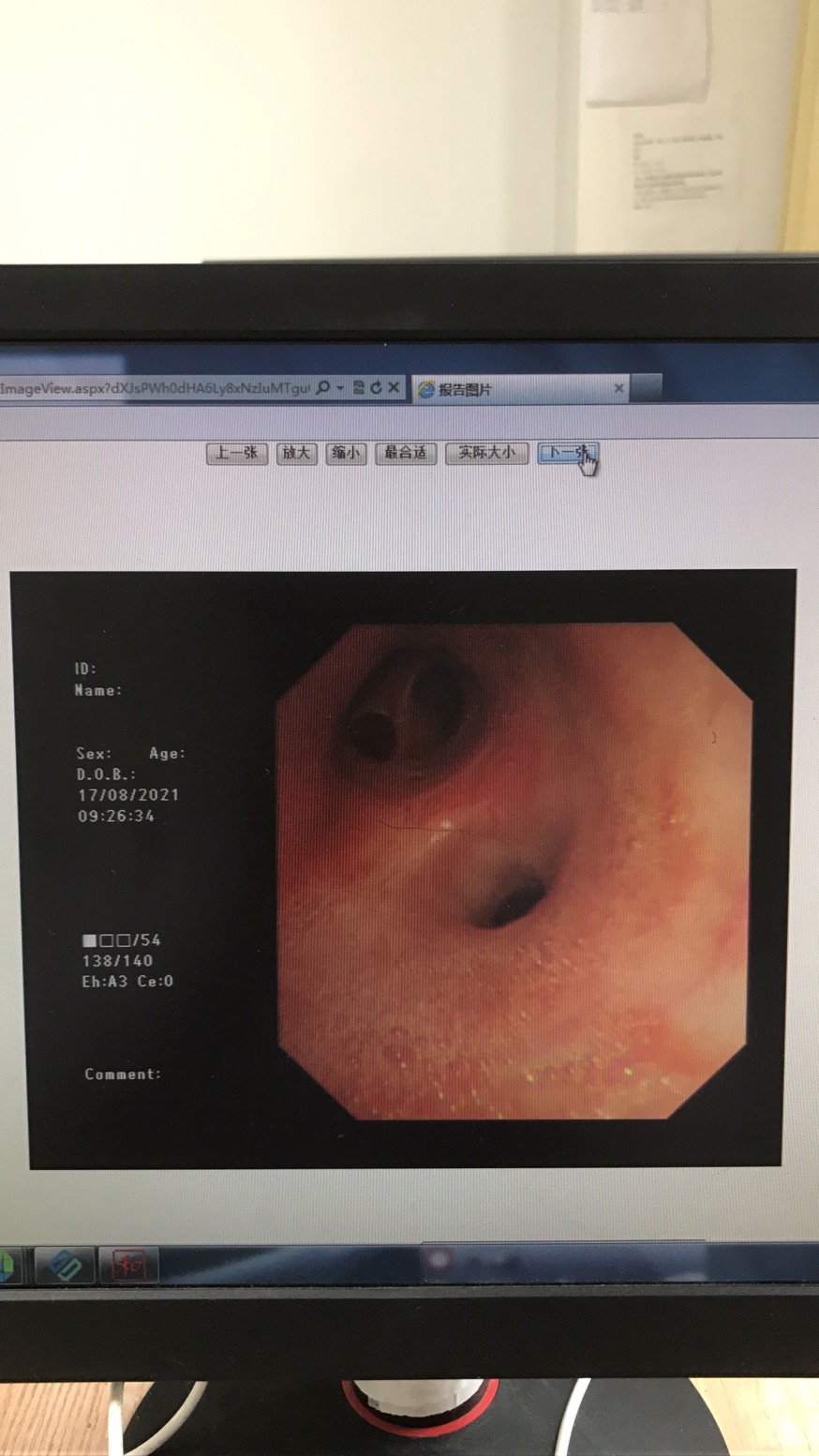

Supplement: Supplementary file 1 [file Image_1.jpeg]

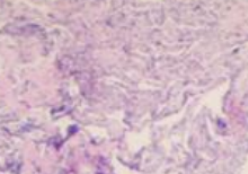

Supplement: Supplementary file 2 [file Image_2.png]

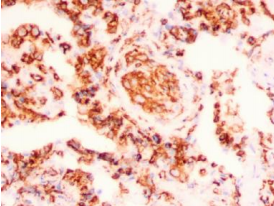

Supplement: Supplementary file 3 [file Image_3.png]

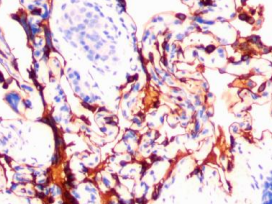

Supplement: Supplementary file 4 [file Image_4.png]
